# Editorial: AI-powered advances in diagnosis and management of movement disorders

**DOI:** 10.3389/fneur.2026.1871403

**Published:** 2026-05-27

**Authors:** Yi-Min Sun, Gao-Wei Xu, Guang-Ming Zhang, Jian-Jun Wu

**Affiliations:** 1 Department of Neurology and National Research Center for Aging and Medicine and National Center for Neurological Disorders, State Key Laboratory of Brain Function and Disorders, Huashan Hospital, Fudan University, Shanghai, China; 2 College of Electronic and Information Engineering, Tongji University, Shanghai, China; 3 McWilliams School of Biomedical Informatics, University of Texas Health Science Center at Houston, Houston, TX, United States

**Keywords:** artificial intelligence, deep phenotyping, diagnosis, digital biomarker, management, movement disorders

Movement disorders are a group of neurological conditions caused by dysfunction of the nervous system that affect the speed, quality, and ease of movement. They can be broadly classified into hyperkinetic (excessive movement) and hypokinetic (slow movement) types. The phenomenology of movement disorders is critical since it acts as a clue to the etiological diagnosis and symptomatic treatments. Traditionally, the assessments of the phenotype are carried out using the scales by the clinical raters. In addition to inter-rater inconsistency, which is always an issue, scale evaluations are time-consuming and limited by clinical settings, all of which affect their objective assessment of symptoms and treatment efficacy, particularly in real-world scenarios requiring continuous monitoring of the disease. Moreover, lots of useful information might be missed in the data collecting by digital materials of video and audio recordings, neuroimaging, electrophysiological signals, wearable sensors if they are analyzed by traditional methods.

Artificial intelligence (AI) based methods, especially the novel data driven models using machine learning and deep learning, showed potentials for interpreting complex data into quantifiable metrics, which have revolutionized medical data analysis and image processing, assisting in deep phenotyping (1), diagnosis (2, 3), identifying digital biomarkers (4), remote screening (5) and monitoring in movement disorders (6).

This Research Topic highlights the recent advances of AI assisted researches in diagnosis and management of movement disorders. The included studies demonstrated the diagnostic tools of Parkinson's disease (PD), non-motor symptoms related digital phenotyping in PD, AI-based measurement of therapeutic effects in PD, and the quantitative predictors in Meige syndrome.

Accurate and convenient early diagnosis is an important issue to address in PD. Joseph Raj et al. developed a multimodal machine learning model for early detection of PD. Based on a spiral drawn by the patients, they used an ensemble approach (ESDRCX) to integrate a meta-ensemble stacking technique that incorporates Decision Tree, Support Vector Machine and Random Forest using quantitative data from handwriting features of the spiral, along with a Convolutional Neural Network (CNN) for images of the spiral. Additionally, the outputs are merged using XGBoost as the meta-learner optimized with Optuna-based Tree structured Parzen Estimator (TPE). The ESDRCX achieves 95.7% accuracy and 87% AUC of PD detection.

Chen et al. proposed a method to classify PD from healthy controls, using voice recordings of the subjects. In their study, three pre-trained deep learning models (convolutional neural networks) were used to extract features from the spectrograms converted from the speech segments, followed by feature fusion. The fusion of two models achieved the highest accuracy of 95.56% and an AUC of 0.99, better than the best single model (92.73% accuracy).

These methods provide a low-cost, non-invasive and a convenient tool for PD diagnosis. They also emphasized the importance of multimodal framework and multi-model integration in the proposed mode, since the different data types and different algorithms attained better model performance.

Non-motor symptoms were important but seemed less enrolled as phenotype in the AI analysis. Here, Serbée et al. investigated the association between hypomimia and sialorrhea in PD by AI base video analysis of a 10-s videos of happy facial expressions and found the moderate correlations between sialorrhea rating scales and features derived from facial regions. The videos were analyzed using AI algorithms to extract key facial landmarks which were then processed into 20 quantitative features representing mouth, eyes, and combined facial regions. Principal Component Analysis, Canonical Correlation Analysis, and bootstrapping were applied. Their study indicated the video-based assessment tools may also be extended to screen for non-motor symptoms, which broadened the scope of digital phenotyping in PD.

AI based approach quantifying motor symptoms first characterized the kinematic effects of levodopa on PD related motor features and found 3 kinematic domains of speed, consistency, timing/scale improved by levodopa, reported by Lange et al. in 2025, describing the effects of treatments unable to be detected by conventional scales (7). In this Research Topic, Su et al. evaluated the effects of levodopa and STN-DBS on limb bradykinesia using an AI-based video analysis by MoDAS (movement dysfunction assessment software). MoDAS is a commercial AI-driven software uses computer vision and deep learning-based pose estimation for the quantitative assessment of movement disorders. Levodopa was superior for improving lower-limb amplitude, while DBS benefited upper-limb speed and amplitude, which was not detected in the conventional scoring by the raters. Their results supporting the fact that AI-based video analysis is a more sensitive and multidimensional assessment than traditional clinical ratings. It revealed treatment specific effects that standard scales missed, which may support personalized treatment decisions in PD.

Beyond these clinical implications, the AI-assisted findings might offer clues of novel mechanisms, for example, the different mechanisms of medication and DBS effect in Su et al.'s study. In another study by Liu et al., they built and validated a clinical prediction nomogram model for Meige syndrome, providing the risk or protective factors and quantifying them into actionable probabilities. It offered a validated approach for early risk stratification and individualized intervention planning, and also gave some clues to the pathogenesis of Meige syndrome.

The unqualified or inconsistent data, patients' privacy protection, poor model generalization, lack of real-world validation or external validation, black-box effect, might be the challenges and concerns of AI use in movement disorders and should be improved in the future (6). Finally, it is important to point out that AI serves as an auxiliary tool for diagnosis and management in movement disorders, but it could not replace the role of a physician currently (8).
